# A Novel Avian Paramyxovirus (Putative Serotype 15) Isolated from Wild Birds

**DOI:** 10.3389/fmicb.2017.00786

**Published:** 2017-05-05

**Authors:** Hyun-Jeong Lee, Ji-Ye Kim, Youn-Jeong Lee, Eun-Kyung Lee, Byoung-Min Song, Hee-Soo Lee, Kang-Seuk Choi

**Affiliations:** ^1^Avian Disease Division, Animal and Plant Quarantine AgencyGimcheon-si, South Korea; ^2^Animal Veterinary Drugs and Biologics Division, Animal and Plant Quarantine AgencyGimcheon-si, South Korea

**Keywords:** avian paramyxovirus, wild duck, UPO wetland, novel serotype, serotype 15

## Abstract

In January 2014, a viral hemagglutinating agent named UPO216 was isolated from fecal droppings of wild birds at the UPO wetland in South Korea during an avian influenza surveillance program. Electron microscopy identified the UPO216 virus as an avian paramyxovirus (APMV). Pathogenicity tests and molecular pathotyping revealed that the virus was avirulent in chickens. The UPO216 virus was assigned to a serological group antigenically distinct from known serotypes of APMV (−1, −2, −3, −4, −6, −7, −8, and −9) by hemagglutination inhibition test, despite showing weak cross-reactivity with APMV-1 and APMV-9. The UPO216 virus RNA genome is 15,180 nucleotides (nts) in length, encodes 3′-N-P(V/W)-M-F-HN-L-5′ in that order, and shows unique genetic characteristics in terms of genomic composition and evolutionary divergence (0.43 or greater from known serotypes of APMV). Phylogenetic analysis revealed that the UPO216 occupies a branch separate from APMV-1, -9, -12, and -13. Serologic surveillance of wild birds (*n* = 880; 15 species, five Orders) detected UPO216-reactive antibodies in 4% (20/494) of serum samples taken from five species of wild duck belonging to the Order *Anseriformes*. In particular, UPO216-specific antibodies showing no cross-reaction with other serotypes of APMV were detected in four species: Eurasian teal (1/36), European wigeon (1/73), mallard (4/139), and Spot-Billed duck (1/137). These results indicate that the UPO216 virus has antigenically and genetically unique characteristics distinct from known serotypes of APMV and likely has been circulating widely in wild duck species of the Order *Anseriformes*. Thus, we propose the UPO216 isolate as a prototype strain of a novel APMV serotype (putative APMV-15).

## Introduction

Avian paramyxoviruses (APMVs) belong to the genus *Abulavirus* within the family *Paramyxoviridae*. The genomes of APMVs contain a non-segmented negative sense single-stranded RNA ranging from 15 to 17 kb in length and encoding at least six proteins, nucleocapsid (N), phospho- (P), matrix (M), fusion (F), hemagglutinin-neuraminidase (HN), and a large polymerase (L). Two additional proteins, V and W, may be produced by an RNA editing event during transcription of the P gene (Miller et al., 2010a; Samuel et al., 2010; Samal, 2011). APMV serotype 6 (APMV-6) contains a small hydrophobic protein (SH) gene between the F and HN genes, which is not found in other APMV serotypes (Chang et al., 2001; Samuel et al., 2010).

APMVs are divided into 14 different serotypes (APMV-1 to −14) according to cross-hemagglutination inhibition (HI) testing (Alexander, 2003) and/or by phylogenetic analysis (Miller et al., 2010a; Terregino et al., 2013; Yamamoto et al., 2015; Thampaisarn et al., 2017). To date, numerous APMVs have been isolated from domestic and wild birds worldwide. Nine known serotypes (APMV-1 to -9) were discovered worldwide before the 1980s, and some APMV serotypes infect domestic poultry. Poultry, especially chickens, are highly susceptible to APMV-1, also known as Newcastle disease virus (NDV), which causes substantial economic losses; however, serotypes APMV-2, −3, −6, and −7 cause only mild respiratory illness and/or egg drop in chickens and turkeys (Suarez et al., 2013).

Since 2005, more than four novel APMV serotypes (APMV −10 to −14) have been isolated from wild bird populations. APMV serotypes −10, −11, and −12 were isolated from Rockhopper penguins (*Eudyptes chrysocome*) in the Falkland Islands in 2007 (Miller et al., 2010a), common snipes (*Gallinago gallinago*) in France in 2010 (Briand et al., 2012), and Eurasian wigeon (*Anas penelope*) in Italy in 2005 (Terregino et al., 2013), respectively. Recently, novel APMVs (putative APMV-13 and -14) were isolated from wild birds around the world during an of avian influenza (AI) surveillance program; species included wild migratory geese in Japan in 2010 (Yamamoto et al., 2015), a white-fronted goose in Ukraine in 2011 (Goraichuk et al., 2016), a white-fronted goose in Kazakhstan in 2013 (Karamendin et al., 2016), and wild ducks in Japan in 2011 (Thampaisarn et al., 2017).

The emergence and worldwide spread of the H5N1 highly pathogenic avian influenza virus (AIV) in poultry and wild birds in the early 2000s spurred the development of AI surveillance programs to better understand the ecology of AIVs in wild birds, especially waterfowl, worldwide (Miller et al., 2010a). This has provided opportunities to better understand the ecology of APMVs, for which wild birds are considered natural hosts (Samal, 2011; Suarez et al., 2013).

Since 2003, Korea has conducted a surveillance program aimed at better understanding the ecology of AIVs in wild birds, live poultry markets, and poultry farms. As part of this program, a non-AIV viral hemagglutinating agent was isolated from wild migratory ducks (species unknown) in the southern region of South Korea in January 2014. Here, we examined the biological properties and genomic features of this isolate to determine whether the virus is a novel APMV.

## Materials and methods

### Virus isolation

A hemagglutinating agent, UPO216, was isolated from fresh wild bird droppings collected at the UPO wetlands (35°33′13.9”N 128°24′50.0”E) in the Gyeongnam province of Korea during an AI surveillance program in 2014 by inoculation into the allantoic cavities of 9–11-day-old specific pathogen-free (SPF) embryonated chicken eggs (ECEs, Valo BioMedia, USA) as previously described (Kang et al., 2010). The isolate was propagated in ECEs and UPO216 was named after the geological location of virus isolation. HA-positive allantoic fluids tested negative for AIV and APMV-1 by reverse-transcription polymerase chain reaction (RT-PCR) (Choi et al., 2012; Lee et al., 2014). The virus titer was measured by end-point titration in ECEs and expressed as the 50% egg infective dose (EID_50_), as calculated by the Reed and Muench method (Reed and Muench, 1938).

### Electron microscopy

For electron microscopy, UPO216 virus contained in allantoic fluid was concentrated by 20–60% (w/v) continuous sucrose gradient centrifugation at 90,000 × g for 1.5 h at 4°C in a Beckman SW41 rotor (Beckman, Germany). Each fraction was collected and tested for HA activity. HA-positive fractions were diluted in phosphate buffered saline (PBS, pH 7.4) and precipitated by centrifugation at 26,000 × g for 6 h at 4°C. The pellet was resuspended in PBS and kept at 4°C until use. Purified virus was adsorbed to 300 mesh copper grids at room temperature. The grids were negatively stained with 2% uranyl acetate and examined under a Hitachi 7100 electron microscope (Hitachi, Japan) at a magnification of × 100,000.

### Virus serotyping

To serotype APMVs, a cross-HI test was performed using a reference panel comprising antigens and chicken anti-sera against representatives of APMV-1 to APMV-9 (except APMV-5; National Veterinary Service Laboratories, USA) using the method outlined in OIE (world organization for animal health) terrestrial manuals (OIE, 2012). Chicken antiserum homologous for the UPO216 virus was prepared by intravenously injecting 3-week-old SPF chickens with purified UPO216 virus (10^9.0^ EID_50_ per dose) as previously described (Miller et al., 2010a). The antigenic relatedness (R) between the two viruses was calculated using the method of Archetti and Horsfall (1951): *R* = (*r*_1_ × *r*_2_)^1/2^, where *r*_1_ is the ratio of the heterologous HI titer to the homologous HI titer for virus 1 and *r*_2_ is the ratio of the heterologous HI titer to the homologous HI titer for virus 2.

### *In vivo* pathogenicity testing

The virulence of the virus was determined by the intracerebral pathogenicity index (ICPI) in day-old SPF chicks and the mean death time (MDT) in 9–11-day-old embryonated SPF ECEs, as previously described (Choi et al., 2012). All procedures were approved and supervised by the Institutional Animal Care & Use Committee of the Animal and Plant Quarantine Agency (QIA).

### Genome sequencing

Viral RNA was extracted from the allantoic fluid using a QIAmp viral RNA mini kit (Qiagen, USA) according to the manufacturer′s instructions. Complementary DNA synthesis was performed using a LaboPass™ cDNA synthesis kit (Cosmogenetech, Korea). A combination of F gene-specific primers and primer walking was used to generate PCR amplicons covering the genome (except for both ends). The sequences of the 3′ and 5′ ends of the genome were amplified using the rapid amplification of cDNA ends (RACE) method (Li et al., 2005). All primer sets are described in Table 1. All PCR amplicons were sequenced using fluorescent dideoxynucleotide terminators and an automated sequencer (ABI 3730XL automated sequencer; Applied Biosystems Inc., USA).

**Table 1:** **Primers used for sequencing of full-length genome of UPO216 virus in this study**.

**Name**	**Sequences (3′ → 5′)**	**Size (bp)**	**Usage**
53F	CGYACGGGTAGAAGGTGTGA	20	Sequensing
1561F	AGGCAACCAAGACCAGGATCAGG	23	Sequensing
1814R	CTTCTACCCGTAYTTTTTTCTA	22	Sequensing
2223F	ATACGGACAGGTGCGAGCTC	20	Sequensing
2921F	AGTGAAGGCTAGTCAGGCGC	20	Sequensing
3257F	TAAGAAAAAATACGGGTAGAAT	22	Sequensing
4138R	TGAAGGGCCGAGAACATC	18	Sequensing
4520R	CTTCTACCCGTGTTTTTTCTAA	22	Sequensing
4541F	TCTGCCCTCCTTTGATAATCCAA	23	Sequensing
4885F	GACCGAAACAGCAGGATTAGTTCAGG	26	Sequensing
4896R	GCTGTTTCGGTCGTTGACTCGTGTAT	26	Sequensing
5274R	TGAATACTGAGTGGACTAAGAGCCGGA	27	Sequensing
5623F	CTTCCCTATGTCTCCAGG	18	Sequensing
6116F	TCTCCTGTGACAGGTAGTAG	20	Sequensing
6654F	YCAAGATGTCRTAGATAGG	19	Sequensing
7668R	CTCCAATGTGCATGACTC	18	Sequensing
8210F	TGATGCCATCGCAGAACCCC	20	Sequensing
8401R	GCYYGCCATGTCCTACCCGT	20	Sequensing
8943F	ACAGCTCCAGCGACATTT	18	Sequensing
9095F	TTGATGTATGCRGATATGAT	20	Sequensing
9773F	ACTTCGACCCAGTCTCAA	18	Sequensing
10524R	ACGATRTATATGTCATCATT	20	Sequensing
10578F	GACAATGATTTCCATATCTG	20	Sequensing
12201R	TTCCTGCTGTTGGGAGCGGT	20	Sequensing
12932R	GATATGGTTGCTGCTATG	18	Sequensing
13788R	GCGGCACATGCAACTCTA	18	Sequensing
14240F	MGAGGRGATATGGAGTGTTA	20	Sequensing
14459R	TAAGCCCTGGGGTGGGTAGA	20	Sequensing
15179R	ACCAAACARAGATTTGGTGA	20	Sequensing
398R1	GTCCAGCTAGGGCAACAT	18	3′RACE
303R2	CTAACTGCCACTCTGAGG	18	3′RACE
14325PF	phospho-AGGTGGTGAGGATGGCGAAG	28	5′RACE
14862F1	GGAGCACCTGCCCAAAATA	19	5′RACE
14652R1	GCTAGTGCACCGCCTTCTT	19	5′RACE
14979F2	TTATCGGGAATGCAATCAAAG	21	5′RACE
14618R2	ATCTAATGGCAGTATCTATGTGG	23	5′RACE

### Phylogenetic analysis

Genome sequences of representative viruses belonging to the family Paramyxoviridae and sequences of each APMV serotype (all available in GenBank) were used for phylogenetic comparison. Editing and sequence analyses were performed using the BioEdit sequence alignment editor (Hall, 1999). Alignment of multiple nt and amino acid (aa) sequence alignments for the complete genome, the F gene, and the HN gene was performed using CLC Genomic Workbench 6.7.2 (CLC bio Aarhus, Denmark). Phylogenetic analysis was performed in MEGA 7.0 (Kumar et al., 2016) using both the maximum parsimony and maximum likelihood methods, with 1,000 bootstrap replicates. The evolutionary distance between and within APMV serotypes was determined using MEGA 7.0 (over 1,000 bootstrap replicates; Kumar et al., 2016).

### Accession numbers

Genome sequences of representative APMV serotypes were retrieved from GenBank public databases and used for the alignments. The GenBank accession numbers are as follows: APMV-1, accession nos. JF950510, AJ88027, and JF893453; APMV-2, accession nos. HM159993, HM159995, and HQ896023; APMV-3, accession nos. EU403085 and EU782025; APMV-4, accession nos. FJ177514 and EU877976; APMV-5, accession no. GU206351; APMV-6, accession nos. JN571486 and GQ406232; APMV-7, accession no. FJ231524; APMV-8, accession no. FJ619036; APMV-9, accession no. EU910942; APMV-10, accession no. HM159995; APMV-11, accession no. JQ886184; APMV-12, accession no. KC333050; APMV-13, accession nos. LC041132 and KX119151; and APMV-14, accession no. KX258200.

### Serological surveillance

A total of 880 serum samples from wild birds in Korea were examined. The serum samples were originally taken from wild birds captured for an AI surveillance program; samples were kept at the AI laboratory, QIA, Korea. The serum samples were obtained from five Orders: *Anseriformes, Charadriiformes, Ciconiiformes, Columbiformes*, and *Passeriformes* covering 15 species. Sera were heat-inactivated at 56°C for 30 min before use in the serologic assay. Serological HI tests employing four HA units of test antigen in V-bottom microtiter plates were performed as described previously (OIE, 2012). The serum samples were first screened for the UPO216 virus isolated in the present study, and positive samples were further tested for cross-reaction with other APMV serotypes. The HI antibody titer was calculated as the reciprocal of the highest serum dilution that completely inhibited 4 HA units of antigens. HI titers ≥8 (3log_2_) were considered positive. All tests were repeated twice.

## Results

### Biological characterization

**Figure 1:**
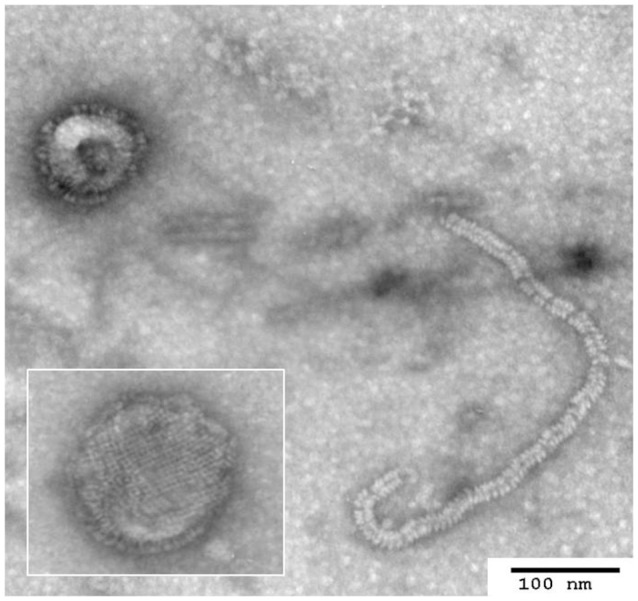
**Electron microscopy analysis of negatively stained APMV isolate UPO216**. Virus particles with surface projections contain nucleocapsids. Herringbone-like structures (nucleocapsids) typical of paramyxovirus are also observed. (Magnification: ×100,000).

The UPO216 virus was successfully propagated in ECEs, and the harvested infective allantoic fluid had a titer of 10^9.3^ EID_50_ per ml and a HA titer of 1,024–2,048 per 25 μl. Electron microscopy revealed that the UPO216 virus particles showed pleomorphic morphology, with a diameter of 80–300 nm. Glycoprotein projections of approximately 10 nm in length were evenly distributed across the envelope of the virus particle. Herringbone-like structures (nucleocapsids) were packaged inside the virion (Figure 1). These characteristics are typical of a paramyxovirus. *In vivo* pathogenicity testing revealed that UPO216 had a MDT index of >120 h and an ICPI-value of 0.00 (Table 2), indicating that it is avirulent in chickens. Serotyping revealed that UPO216 had a high HI titer (1:1,024) for homologous chicken antiserum (as expected), but low cross-reactivity (HI titers of ≤ 1:128) with several APMV serotypes, including APMV-1 and -9. The R between UPO216 virus and APMV serotypes was then calculated based on the HI titers obtained from the cross-HI test (Table 3). The UPO216 virus showed an *R*-value of <0.05 for all APMV serotypes except APMV-1 (*R* = 0.088) and APMV-9 (*R* = 0.125). The higher *R*-values for APMV-1 and APMV-9 were due to weak cross-reactions in the cross-HI test.

**Table 2:** **Prototype APMVs and UPO/14 and their pathogenicity in chickens**.

**APMVs**	**Isolate**	**F cleavage**	**MDT (h)**	**ICPI**	**References**
UPO216	UPO216	LVQAR↓L	>120	0.00	This study
APMV-1	LaSota (avirulent)	GRQGR↓L	112	0.00	Kim et al., 2012
	BC (virulent)	RRQKR↓F	58	1.55	Kim et al., 2012
APMV-2	California/Yucaipa/56	KPASR↓F	>168	0.00	Subbiah et al., 2010
	Bangor/1973	TLPSAR↓F	>168	0.00	Subbiah et al., 2010
APMV-3	Netherlands/449/75	RPRGR↓L	112	0.39	Kumar et al., 2010
	Wisconsin/1968	RPSGR↓L	>168	0.00	Kumar et al., 2010
APMV-4	Hong Kong/D3/1975	DIQPR↓F	>144	0.00	Kumar et al., 2010
APMV-5	Kunitachi/1974	KRKKR↓F	>144	0.00	Kumar et al., 2010
APMV-6	Hong Kong/199/1977	APEPR↓L	>144	0.00	Kumar et al., 2010
APMV-7	TN/4/1975	LPSSR↓F	>144	0.00	Kumar et al., 2010
APMV-8	DE/1053/1976	YPQTR↓L	>144	0.00	Kumar et al., 2010
APMV-9	New York/22/78	IREGR↓I	>144	0.00	Kumar et al., 2010
APMV-10	Falkland Islands/324/2007	KPSQR↓I	>90	0.00	Miller et al., 2010a
APMV-11	France/100212/2010	SGTKR↓F	NA	NA	Briand et al., 2012
APMV-12	Italy/3920-1/2005	GREPR↓L	NA	0.45	Terregino et al., 2013
APMV-13	Shimane/67/2000	VRENR↓L	>120 h	0.0	Yamamoto et al., 2015
APMV-14	Japan/11OG0352/2011	TREGK↓L	NA	0.0	Thampaisarn et al., 2017

**Table 3:** **Antigenic relatedness of avian paramyxoviruses as determined by Archetti and Horsfall calculations based on HI test results**.

**Virus**	**Antiserum**								
	**UPO216**	**APMV-1**	**APMV-2**	**APMV-3**	**APMV-4**	**APMV-6**	**APMV-7**	**APMV-8**	**APMV-9**
UPO216	1.000	0.098^a^	0.008	0.044	0.016	0.003	0.044	0.031	0.125
APMV-1		1.000	0.001	0.006	0.004	0.001	0.004	0.001	0.044
APMV-2			1.000	0.011	0.008	0.022	0.031	0.008	0.004
APMV-3				1.000	0.016	0.006	0.044	0.008	0.044
APMV-4					1.000	0.022	0.016	0.008	0.016
APMV-6						1.000	0.031	0.004	0.006
APMV-7							1.000	0.011	0.044
APMV-8								1.000	0.011
APMV-9									1.000

a *Numbers represent R-values calculated from the cross-HI results using the method of Archetti and Horsfall (1951). Homologous viruses give values of 1*.

### Complete genome sequence

The nt sequence of the UPO216 virus was compiled from the sequences of 28 overlapping cDNA clones covering the entire genome. The UPO216 genome comprises 15,180 nts (accession no. KY511044). This length conforms to the “rule of six,” which plays an important role in the replication of paramyxoviruses (Kolakofsky et al., 1998).

A genome-wide BLASTN search (http://blast.ncbi.nlm.nih.gov/Blast.cgi) showed that UPO216 was closely related with known APMVs, especially with the highest identity with NDV strain 08-004 (accession no FJ794269.1; 75% identity) for N protein gene (positions 1–1,326) and with NDV strain BHG/Sweden/94 (accession no GQ918280; 72% identity) for L gene (positions 9,415–12,945). This indicates that the UPO216 virus might be an APMV.

**Figure 2:**
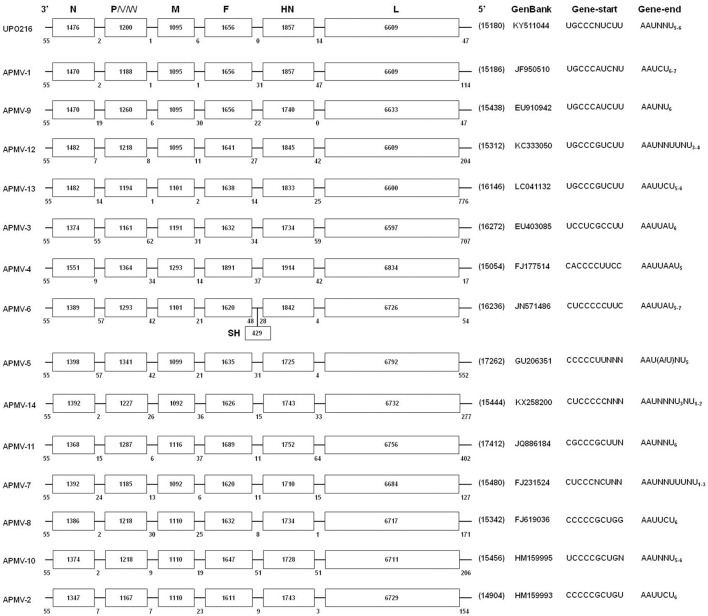
**Schematic diagram showing the genome composition of APMV isolate UPO216 and a comparison with known serotypes of APMV**. Individual genes are indicated by rectangles, with the gene names given at the top. The length of the gene [in nucleotides (nt)] is shown in each box, and the lengths of the 3′ leader, 5′ trailer, and IGS sequences are shown below the line. The genome size, accession number, and gene start and end motifs for each APMV serotypes are shown to the right.

The genome of UPO216 contains six non-overlapping transcriptional units in the following order: 3′-leader-NP-P-M-F-HN-L-trailer-5′ (Figure 2). Two additional proteins, V (245 aa) and W (140 aa), may arise during transcription of the P gene due to a putative RNA editing site (nt positions 2,292–2,300) in the viral genome. This occurs via addition of a single G residue to the editing site to yield a predicted V protein and the addition of two G residues to yield a predicted W protein, as is the case for APMV-1 (Steward et al., 1993). However, SH gene, that is found in APMV-6 (Chang et al., 2001), is not present between the F and HN genes. The 3′ leader sequence is 55 nt in length; this length is conserved among most APMV serotypes (Samuel et al., 2010; Samal, 2011). The length of the trailer at the 5′ end is 47 nt, the same as that in APMV-9 (Samuel et al., 2009; Figure 2). The first 12 nt of the leader sequence (3′-UGGUUU GUCUCU-5′) and the last eight nt of the 5′ trailer sequence (5′-ACCAAA CAAAGA-3′) show a high degree of homology (91.7%). The conserved sequences for the gene start (GS) and gene end (GE) of the UPO216 virus are UGC_3_A/CUCUU and AAUNC/UU_5−6_, respectively. The length of the intergenic region sequences ranges from 0 to 14 nt. The F protein cleavage sites within UPO216 possess the following unique aa sequence: ^110^L-V-Q-A-R-L^115^ (Table 2).

Comparison of the complete consensus sequences for the UPO216 genome with those of known APMV serotypes revealed that UPO216 is most closely related to APMV-1 (64.0% identity), followed by APMV-9 (56.4%), APMV-12 (52.9%), and APMV-13 (50.9%) (Table 4). UPO216 shows low homology with other APMV serotypes (37.0–41.1%).

**Table 4:** **Percentage nucleotide identity of complete genome sequences of APMVs representing groups APMV-1 to APMV-14**.

**APMV serotype**	**1**	**2**	**3**	**4**	**5**	**6**	**7**	**8**	**9**	**10**	**11**	**12**	**13**	**14**	**UPO216**
1															
2	41.2														
3	36.6	36.2													
4	38.0	37.5	44.3												
5	37.6	40.8	35.6	35.0											
6	39.4	42.9	35.1	36.3	43.8										
7	41.1	47.2	36.6	37.9	41.9	43.3									
8	40.9	55.5	36.3	37.6	41.2	43.2	48.6								
9	56.7	40.3	36.6	37.7	37.2	39	41.0	41.0							
10	41.0	54.1	35.9	38	41.2	44.2	47	54.4	41.6						
11	37.1	41.2	36	34.8	51.2	40.2	42.3	41.7	37.4	42.1					
12	53.5	40.2	36.3	37.9	37.4	38.7	40.9	40.6	52.5	40.7	37.5				
13	50.8	39	38.2	36.3	37.8	37.7	40.4	39.9	49.4	38.9	38.3	58.2			
14	40.2	44.6	36.5	37.3	44.3	49.9	44.6	45.8	40.2	45.6	41.0	40.3	39.4		
UPO216	64.0	40.8	36.2	37.9	37.0	38.5	41.1	41.0	56.4	40.8	37.5	52.9	50.9	40.4	

*The numbers of the closest identity (%) between viruses of the two APMV groups are shown*.

### Phylogenetic analysis

**Figure 3:**
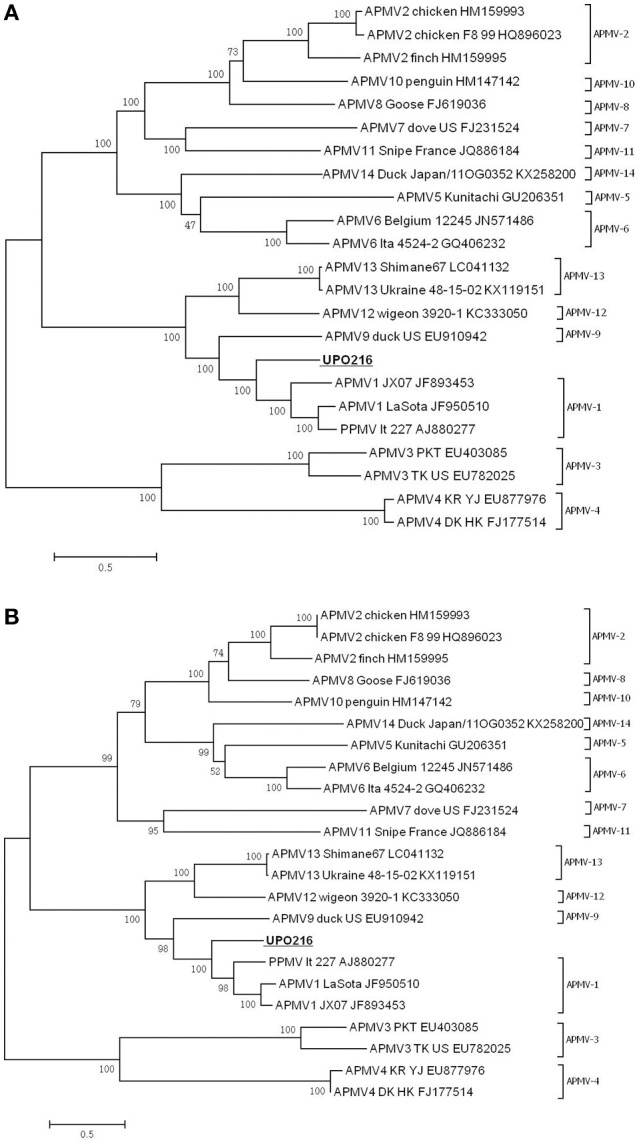
**Phylogenetic analysis of the UPO216 virus based on the complete sequence of the genome (A)** and F gene **(B)**.

A phylogenetic tree was constructed based on alignment of the complete nt sequences of the UPO216 genome and F gene with those of representative APMV serotypes (Figure 3). Phylogenetic analysis based on the complete sequences revealed that UPO216 formed a separate phylogenetic group along with APMV-1, -9, -12, and -13 (Figure 3A). Within this group, UPO216 is more closely related to APMV-1 and APMV-9 than to APMV-12 and APMV-13. APMV-3 viruses are closely related to APMV-4 viruses, APMV-5 viruses to APMV-6 and APMV-14 viruses, and APMV-8 to APMV-10 and APMV-2 viruses. Similar results were observed for phylogenetic analyses based on F gene sequences (Figure 3B).

### Evolutionary relatedness

To determine the genetic distance between the UPO216 and known APMV serotypes, we calculated the inter-serotype and intra-serotype evolutionary distances among APMV serotypes based on the sequences of complete genome (Table 5). At the genome level, estimates of the average inter-serotypic distance ranged from 0.43 to 1.62. The lowest inter-serotypic distance was between APMV-1 and UPO216 (0.43), followed by APMV-12 and APMV-13 (0.54), APMV-9 and UPO216 (0.64), APMV-2 and -10(0.69), and APMV-8 and APMV-10(0.69). When evolutionary distance was calculated with known serotypes of APMV, UPO216 was relatively close to APMV-1(0.43), APMV-9(0.64), APMV-12(0.78) and APMV-13(0.77), compared to other APMV serotypes (1.35–1.55). Estimates of intra-serotypic distances for APMV-1, -2, -3, and -6 were 0.21, 0.21, 0.30, and 0.26, respectively.

**Table 5:** **Estimated evolutionary divergence between and within APMV groups in terms of complete genome sequences (below diagonal) and HN gene amino acid sequences (above diagonal)**.

**APMV serotypes**	**1**	**2**	**3**	**4**	**5**	**6**	**7**	**8**	**9**	**10**	**11**	**12**	**13**	**14**	**UPO216**
1	**0.21**	0.64	0.64	0.64	0.65	0.68	0.62	0.65	0.37	0.65	0.87	0.41	0.44	0.64	0.27
2	1.36	**0.21**	0.66	0.67	0.56	0.57	0.58	0.50	0.68	0.49	0.85	0.66	0.65	0.59	0.64
3	1.49	1.50	**0.30**	0.58	0.66	0.68	0.66	0.68	0.63	0.66	0.87	0.63	0.61	0.63	0.65
4	1.54	1.54	1.13	**0.06**	0.69	0.67	0.64	0.67	0.63	0.69	0.89	0.63	0.61	0.66	0.63
5	1.46	1.29	1.58	1.62	**NA**	0.41	0.58	0.58	0.68	0.57	0.87	0.67	0.66	0.46	0.66
6	1.37	1.15	1.50	1.57	0.96	**0.26**	0.56	0.60	0.68	0.58	0.88	0.66	0.66	0.48	0.69
7	1.39	1.13	1.51	1.54	1.25	1.18	**NA**	0.59	0.64	0.59	0.87	0.65	0.61	0.56	0.62
8	1.37	0.71	1.50	1.55	1.28	1.14	1.09	**NA**	0.65	0.49	0.86	0.67	0.66	0.56	0.63
9	0.64	1.35	1.50	1.55	1.46	1.36	1.39	1.34	**NA**	0.64	0.87	0.41	0.44	0.65	0.37
10	1.34	0.69	1.51	1.56	1.23	1.12	1.12	0.69	1.35	**NA**	0.89	0.63	0.64	0.59	0.64
11	1.38	1.03	1.50	1.53	1.17	1.12	0.88	1.04	1.36	1.04	**NA**	0.86	0.87	0.89	0.86
12	0.80	1.37	1.52	1.53	1.44	1.38	1.38	1.35	0.81	1.37	1.36	**NA**	0.37	0.65	0.42
13	0.79	1.39	1.46	1.52	1.48	1.37	1.38	1.35	0.84	1.39	1.38	0.54	**0.02**	0.64	0.41
14	1.39	1.21	1.50	1.58	1.00	0.87	1.21	1.14	1.37	1.13	1.15	1.36	1.38	**NA**	0.64
UPO216	0.43	1.36	1.53	1.55	1.49	1.37	1.36	1.35	0.64	1.37	1.36	0.78	0.77	1.35	**NA**

*The numbers of base substitutions per site from between sequences are shown below the diagonal. The number of amino acid differences per site and above the diagonal. Analyses were conducted using the Maximum Composite Likelihood model in MEGA7.0 (Kumar et al., 2016)*.

*Numbers in bold represent the highest divergence based on the complete genome sequences, calculated for different viruses within a group*.

*NA, not applicable (fewer than 10 sequences were available)*.

### Serological surveillance of wild birds

The 880 serum samples examined herein were first tested for antibodies specific for UPO216 in the HI test. Of these, 20 sera reacted with UPO216 (Table 6). These seropositive results were observed in sera obtained from wild ducks (Eurasian teal, European wigeon, mallard, Spot-Billed duck, and mandarin duck) belonging to the Order *Anseriformes*, but not in sera obtained from birds belonging to the Orders *Charadriiformes, Ciconiiformes, Columbiformes*, and *Passeriformes*. When seropositive sera (*n* = 20) were further tested in HI tests based on antigens derived from other serotypes of APMV, seven [Eurasian teal (*n* = 1), mallard (*n* = 4), European wigeon (*n* = 1), and Spot-Billed duck (*n* = 1)] did not cross-react with other APMV serotypes tested, and HI titers for UPO216 ranged from 3log_2_ to >6log_2_. The HI titers of the other 13 sera were the same (or higher) for APMV-1 as for UPO216.

**Table 6:** **Serological tests of UPO216-reactive HI antibodies in serum from wild birds in Korea**.

**Order**	**Species**	**No. tested**	**No. positive (%)**	**HI titer (log_2_)**			
				**3**	**4**	**5**	**≥6**
*Anseriformes*	Anas crecca (Eurasian Teal)	36	2 (5.5)	2 (1)^†^			
	Anas Penelope (European Wigeon)	73	1 (1.4)		1 (1)		
	Anas platyrhynchos (Mallard)	139	6 (4.3)	1(0)	2 (1)	1 (1)	2 (2)
	Anas poecilorhyncha (Spot-Billed Duck)	137	3 (2.2)	2 (0)	1 (1)		
	Aix galericulata (Mandarin duck)	109	8 (7.3)	4 (0)	1 (0)	2 (0)	1 (0)
*Charadriiformes*	Larus argentatus (Herring gull)	4	0				
	Larus crassirostris (Black-tailed gull)	45	0				
*Ciconiiformes*	Ardea cinerea (Gray heron)	13	0				
	Egretta garzetta (Little egret)	81	0				
	Mesophoyx intermedia (Intermediate Egret)	149	0				
	Nycticorax nycticorax (Black-crowned night heron)	13	0				
*Columbiformes*	Columba rupestris (Dove)	61	0				
*Passeriformes*	Cyanopica cyanus (Azure-winged magpie)	9	0				
	Hirundo rustica (Barn swallow)	4	0				
	Passer montanus (Eurasian Tree Sparrow)	7	0				
Total		880	20 (2.3)	9	5	3	3

† *Numbers in parentheses represent the number of sera showing no cross-reactivity with other serotypes of APMVs*.

## Discussion

Wild birds, especially waterfowl, are considered the natural reservoir for a variety of avian viruses (e.g., AIVs) with the potential to cause infectious diseases in poultry. In recent years, several novel APMVs belonging to APMV serotypes 10–14 were reported in wild birds and penguins around the world (Miller et al., 2010a; Briand et al., 2012; Terregino et al., 2013; Yamamoto et al., 2015; Thampaisarn et al., 2017), implying the presence of additional, as-yet-unreported, novel APMVs in wild birds.

Here, we isolated a hemagglutinating virus (UPO216) from a wild bird fecal sample of unknown species during the 2013/2014 winter season (January 2014) at the UPO wetlands, one of the largest inland wetlands in South Korea. This wetland receives thousands of overwintering migratory birds (mainly ducks and geese) from Arctic regions, starting from late fall. Electron microscopy identified UPO216 as a paramyxovirus, characterized by pleomorphic enveloped virions, projections on the viral envelope, and a “herring-bone” nucleocapsid. Fortunately, UPO216 is unlikely to cause clinical disease in chickens since *in vivo* pathogenicity tests and molecular pathotypic analyses showed that it is avirulent in this species.

Notably, the UPO216 virus in this study has biologically and genetically unique characteristics distinct from known serotypes of APMV reported so far. First, the cross-HI test showed that UPO216 is antigenically distinct from other known serotypes of APMV, although it did cross-react weakly with APMV-1 (*R* = 0.088) and APMV-9 (*R* = 0.125). Such weak cross-reactivity between APMVs is not uncommon (e.g., between APMV-1 and APMV-12 (*R* = 0.125); Terregino et al., 2013). Second, the genomic features of UPO216 are unique among the 14 APMV serotypes examined (Figure 2). Such features include the sequence and length of the complete genome, the transcriptional units, and the non-translated regions (i.e., GS, GE, intergenic, leader, and trailer sequences). UPO216 also harbors a unique cleavage site within the F protein (aa sequence LVQAR↓L), which is distinct from that in known serotypes of APMV. Third, estimates of inter-serotype evolutionary distances based on the nucleotide of the full-length genome revealed that UPO216 was relative close to APMV-1(0.43), APMV-9(0.64), APMV-12(0.78), and APMV-13(0.77), which clustered with UPO216 in phylogenetic analysis. However, the values of the evolutionary divergence were lower than those of the intra-serotypic distances of APMV-1, −2, −3, and −6 which genetic diversity within these serotypes has been reported (Alexander, 2003; Miller et al., 2010b; Suarez et al., 2013). Considering the unique characteristics mentioned above, UPO216 appears to be a novel serotype that evolved from a common ancestor and now occupies a separate branch from APMV-1, −9, −12, and −13. Thus, we propose that the UPO216 virus be tentatively classified as a new serotype of APMV (named APMV-15). If this is confirmed to be the case, then the UPO216 virus can be officially named APMV-15/WB/Kr/UPO216/2014.

The species of wild birds from which the UPO216 virus was originally isolated was not identified at the time of sampling, since virus isolation was conducted from fresh duck droppings in the UPO wetlands. To identify wild bird species that may act as a natural reservoir for the UPO216 virus, we conducted serological surveillance of a variety of wild birds in Korea, including *Anseriformes, Charadriiformes, Ciconiiformes, Columbiformes*, and *Passeriformes*. UPO216-reactive antibodies were detected only in *five* ducks species (Eurasian teal, European wigeon, mallard, Spot-Billed duck, and mandarin duck) belonging to the Order *Anseriformes*, although the frequency of sero-positivity in wild ducks was relatively low (4.0%, 20/494). The majority of UPO216-reactive sera (13/20) from wild ducks (particularly mandarin duck) also reacted with APMV-1 (NDV), which showed HI titers that were the same or greater than those of UPO216, indicating that sero-positive results could be due to mixed infection with APMV-1 and the UPO216 virus, or to a cross-reaction with APMV-1. UPO216-specific antibodies (i.e., showing no cross-reaction with other APMV serotypes) were detected in serum samples (*n* = 7) from four species: Eurasian teal (1/36), European wigeon (1/73), mallard (4/139), and Spot-Billed duck (1/137). In particular, UPO216-specific antibodies with a HI titer of 5log_2_ or greater were detected in mallard ducks. Taken together, these results suggest that at least five species of wild duck (particularly mallard) belonging to the Order *Anseriformes* might be the natural reservoir for the UPO216 virus in Far Eastern countries, including Korea. Further virus isolation and/or serological surveillance studies in wild ducks, especially migratory birds, are needed if we are to better understand the genetic diversity and ecology of the UPO216 virus.

## Author contributions

Conceived and designed the experiments: HJL, JK, KC; Performed the experiments: HJL, JK, EL, BS; Supervised and discussed the experiments and data: KC, YL, HSL; Wrote the manuscript: HJL and KC.

## Funding

This research was supported by a grant from the QIA (no. N-1543084-2015-99-02), Republic of Korea. The funders played no role in the study design, data collection and analysis, the decision to publish, or preparation of the manuscript.

### Conflict of interest statement

The authors declare that the research was conducted in the absence of any commercial or financial relationships that could be construed as a potential conflict of interest.
